# Using Mobile Health Technology to Deliver a Community-Based Closed-Loop Management System for Chronic Obstructive Pulmonary Disease Patients in Remote Areas of China: Development and Prospective Observational Study

**DOI:** 10.2196/15978

**Published:** 2020-11-25

**Authors:** Ning Deng, Juan Chen, Yiyuan Liu, Shuoshuo Wei, Leiyi Sheng, Rong Lu, Zheyu Wang, Jiarong Zhu, Jiye An, Bei Wang, Hui Lin, Xiuyan Wang, Yumin Zhou, Huilong Duan, Pixin Ran

**Affiliations:** 1 Ministry of Education Key Laboratory of Biomedical Engineering College of Biomedical Engineering and Instrument Science Zhejiang University Hangzhou China; 2 Engineering Research Center of Cognitive Healthcare of Zhejiang Province (Sir Run Run Shaw Hospital) Zhejiang University Hangzhou China; 3 Department of Pulmonary and Critical Care Medicine General Hospital of Ningxia Medical University Yinchuan China; 4 State Key Laboratory of Respiratory Disease National Clinical Research Center for Respiratory Diseases, Guangzhou Institute of Respiratory Health First Affiliated Hospital of Guangzhou Medical University Guangzhou China

**Keywords:** COPD, mobile health technology, closed-loop care pathway, chronic disease management, exacerbations

## Abstract

**Background:**

Mobile health (mHealth) technology is an increasingly recognized and effective method for disease management and has the potential to intervene in pulmonary function, exacerbation risk, and psychological status of patients with chronic obstructive pulmonary disease (COPD).

**Objective:**

This study aimed to investigate the feasibility of an mHealth-based COPD management system designed for Chinese remote areas with many potential COPD patients but limited medical resources.

**Methods:**

The system was implemented based on a tailored closed-loop care pathway that breaks the heavy management tasks into detailed pieces to be quantified and executed by computers. Low-cost COPD evaluation and questionnaire-based psychological intervention are the 2 main characteristics of the pathway. A 6-month prospective observational study at the community level was performed to evaluate the effect of the system. Primary outcomes included changes in peak expiratory flow values, quality of life measured using the COPD assessment test scale, and psychological condition. Acute exacerbations, compliance, and adverse events were also measured during the study. Compliance was defined as the ratio of the actual frequency of self-monitoring records to the prescribed number.

**Results:**

A total of 56 patients was enrolled; 39 patients completed the 6-month study. There was no significant difference in the mean peak expiratory flow value before and after the 6-month period (366.1, SD 106.7 versus 313.1, SD 116.6; *P*=.11). Psychological condition significantly improved after 6 months, especially for depression, as measured using the Patient Health Questionnaire-9 scale (median 6.0, IQR 3.0-9.0 versus median 4.0, IQR 0.0-6.0; *P*=.001). The COPD assessment test score after 6 months of intervention was also lower than that at the baseline, and the difference was significant (median 4.0, IQR 1.0-6.0 versus median 3.0, IQR 0.0-6.0; *P*=.003). The median overall compliance was 91.1% (IQR 67%-100%). In terms of acute exacerbation, 110 exacerbations were detected and confirmed by health care providers (per 6 months, median 2.0, IQR 1.0-5.0). Moreover, 72 adverse events occurred during the study, including 1 death, 19 hospitalizations, and 52 clinic visits due to persistent respiratory symptoms.

**Conclusions:**

We designed and validated a feasible mHealth-based method to manage COPD in remote Chinese areas with limited medical resources. The proposed closed-loop care pathway was effective at the community level. Proper education and frequent communication with health care providers may encourage patients’ acceptance and use of smartphones to support COPD self-management. In addition, WeChat might play an important role in improving patient compliance and psychological distress. Further research might explore the effect of such systems on a larger scale and at a higher evidence level.

## Introduction

Chronic obstructive pulmonary disease (COPD) is one of the most prevalent chronic diseases in China. According to the latest national survey, approximately 99.9 million Chinese adults aged ≥20 years have spirometry-defined COPD [1]. Pulmonary function tests (PFTs) are essential for the diagnosis of COPD [2,3]. However, only 12% of Chinese patients with COPD can receive full PFTs [1], and even fewer can receive full PFTs in remote areas. For example, the Ningxia Hui Autonomous Region is a relatively remote area in China with a population of over 6 million people. The prevalence of COPD in Ningxia is 8.9% in people aged ≥40 years [4]. Only 23.6% of COPD cases are diagnosed, and only 23.3% of COPD cases are treated [4].

Moreover, most COPD patients are unaware of their condition, and few receive follow-ups from doctors. Once diagnosed with COPD, effective management should be performed based on an individualized assessment to improve prognosis [5], consisting of long-term self-management by patients and timely intervention from doctors [6]. Mobile health (mHealth) has become a promising tool for the management of chronic diseases by health care providers [7]. Furthermore, patients are willing to use smartphones to manage chronic diseases [8-10].

Several studies have explored the feasibility of an mHealth-based intervention for COPD management in patients during the stable phase. Farmer et al [11] performed a randomized controlled trial with an intervention group using an internet-linked, tablet computer–based system to manage COPD. van der Heijden et al [12] developed a mobile system to predict and detect exacerbation using an oximeter and spirometer. Zhang et al [13] implemented a smartphone-based Internet of Things system that provided medication reminders, data collection, health education, and communication for COPD self-management. This project offered PFT in community centers in Shanghai. These studies indicate that mHealth systems have the potential to help patients effectively manage COPD. However, most of these studies assumed that patients were in an ideal environment with portable devices and abundant community health care resources, which may not be applicable in remote Chinese areas. 

In areas with decreased resources such as Ningxia that have many potential COPD patients but not enough medical resources, the aforementioned pattern of mHealth-based interventions may not work. First, spirometry is a complex and not readily available test in Chinese remote areas. COPD patients in these areas need a simpler and low-cost approach to help monitor pulmonary function. Second, health care providers in remote areas might not perform a guideline-based, comprehensive intervention, which results in a significant gap between standards of care and medical practice [14]. Third, during long-term management, depression and anxiety are common in COPD patients [15-17] and are strongly related to rehospitalization and exacerbation [18-20]. Therefore, appropriate psychological intervention is an essential preventive strategy. Moreover, COPD patients in remote areas have poor knowledge about their disease, thus leading to low self-management ability. Health care providers need to communicate more with patients to assess their condition and guide treatment.

To address these problems, we propose a pilot closed-loop care pathway that transforms the core content of long-term COPD management into a form that can be recognized and executed by the computer. The 2 main characteristics of the care pathway are (1) COPD evaluation combining peak expiratory flow (PEF) with the COPD assessment test (CAT) and (2) psychological intervention based on multiple scales. Our primary objective was to determine whether the introduction of an mHealth system based on the proposed care pathway could improve the COPD-specific health status of patients, such as pulmonary function, quality of life (QoL), and psychological conditions, in remote areas of China. Our secondary objectives were to determine if there were changes in exacerbations, hospitalizations, or clinic visits after implementation of the mobile app.

## Methods

### Study Design and Patient Recruitment

We conducted a 6-month prospective observational study to explore the effect of the system in a community-based primary care setting. The study was performed in Yinchuan, Ningxia Hui Autonomous Region, China. Patient recruitment was performed between September 2017 and June 2018. The patients were recruited from outpatient clinics of the General Hospital of Ningxia Medical University and its affiliated hospitals throughout Ningxia. Inclusion criteria included (1) age ≥40 years, (2) confirmed diagnosis of COPD according to the Global Initiative for Chronic Obstructive Lung Disease (GOLD) guidelines [5], and (3) access to a smartphone. Exclusion criteria included (1) diagnosis of other lung diseases with similar symptoms or any other disease-causing changes in pulmonary function, (2) significant mental health disorder, (3) visual or aural disorders, (4) no home internet access, and (5) involvement in another study.

Patients meeting the criteria and willing to participate in the study signed the informed consent forms. Ethical approval was granted by the Ethics Committee for the Conduct of Human Research at the General Hospital of Ningxia Medical University (NXMU-GH-2017-273). Baseline data, including demographics, clinical characteristics, and primary outcomes, were first collected through laboratory examination and face-to-face interviews. Patients were instructed on how to install and use the app at the same time. Moreover, we provided a peak flow meter for each patient and ensured that they could correctly use it themselves.

After enrollment, patients started self-management using the app, and health care providers supervised patients through the website. In this study, the health care providers consisted of physicians from the hospital and general practitioners from community health centers. General practitioners were responsible for daily management, whereas physicians were responsible for the treatment of acute exacerbations and professional support. All management procedures followed the proposed closed-loop care pathway. Follow-up was the main intervention for health care providers. Generally, follow-up was conducted every 2 weeks. In the event of a warning or low compliance, an additional follow-up was arranged. Telephone was the traditional tool for follow-up; however, sometimes it is inefficient because patients may not be able to conveniently answer the call or may not want to be disturbed. Therefore, we introduced WeChat, the most popular instant messaging app in China, as an alternative follow-up method for patients.

Furthermore, a WeChat chat group including all patients and 2 health care providers was formed during the study to facilitate communication. Patients needed to login to WeChat with a password, and they could only be invited by the doctors to join the group chat. Only common questions with no sensitive medical information were included in the group. Patients could chat privately with the doctor on WeChat regarding private information.

### Closed-Loop Care Pathway Design

**Figure 1:**
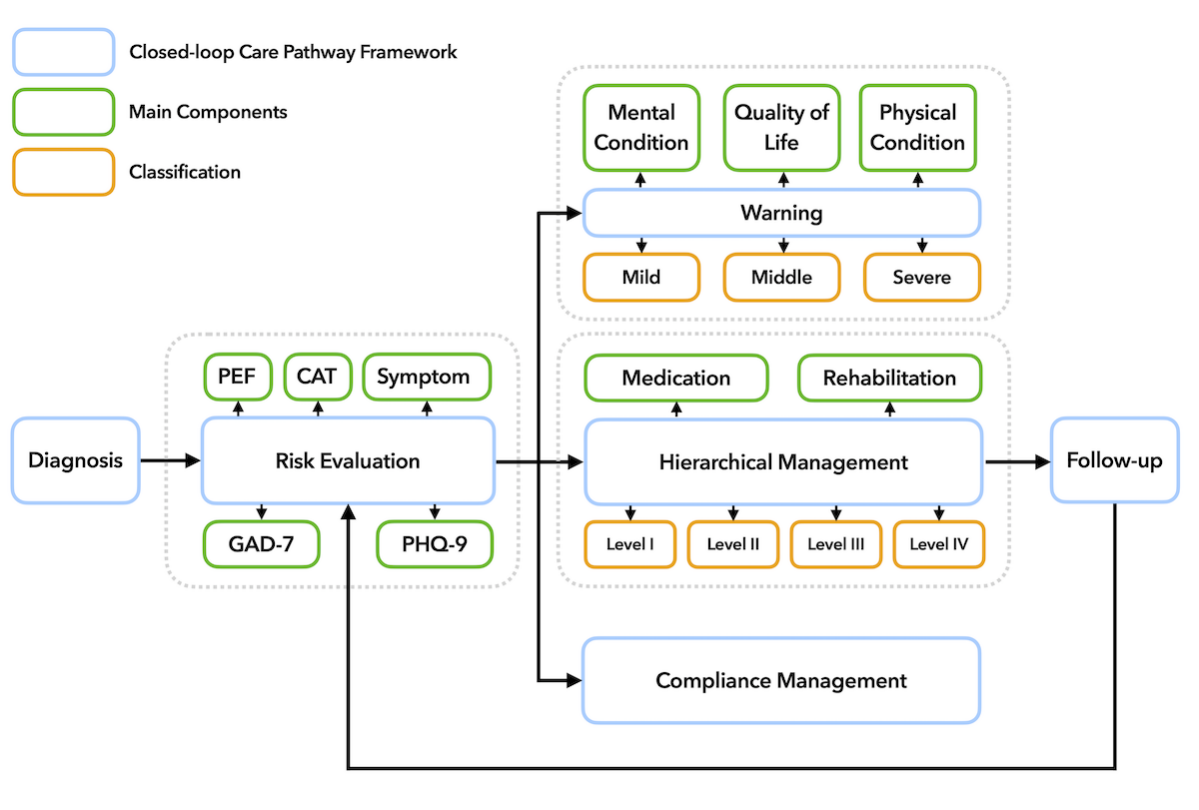
Flow diagram of the proposed care pathway. CAT: Chronic Obstructive Pulmonary Disease Assessment Test; GAD-7: Generalized Anxiety Disorder-7; PEF: peak expiratory flow; PHQ-9: Patient Health Questionnaire-9.

The care pathway mainly included risk evaluation, hierarchical management, follow-up, warning, and compliance management, as shown in Figure 1. Following this pathway, patients were first evaluated and classified into different levels, and then different management strategies including medication and pulmonary rehabilitation were provided. Health care providers performed regular follow-ups with patients to track their health status. Patients’ daily self-monitoring data and compliance were evaluated as well to detect abnormal conditions. A detailed description of each module can be found in Multimedia Appendix 1.

Because of the complexity of spirometry and the lack of professional portable device support in Chinese remote areas, we chose a simpler indicator suitable for home-based management, the PEF, which is the maximum flow achieved during an expiration delivered with maximum force starting from the level of maximal lung inflation [21]. Studies have shown that a PEF rate <80% of predicted may be a good indicator for detecting patients with COPD [22]. Increased variability in PEF could be considered an early index of COPD development [23]. Furthermore, COPD is known to impact patients beyond just dyspnea [5]. Therefore, the CAT, a simple and validated instrument to assess the impact of COPD in routine life [24], is strongly recommended by GOLD. In addition, to monitor patients’ psychological state, patients completed the Patient Health Questionnaire-9 (PHQ-9) scale [25] for depression and Generalized Anxiety Disorder-7 (GAD-7) scale [26] for anxiety. The participants were asked to log their PEF value (the highest value of 3 repeated measurements), CAT, GAD-7, and PHQ-9 scales once every 2 weeks over the 6-month period.

### mHealth System Description

**Figure 2:**
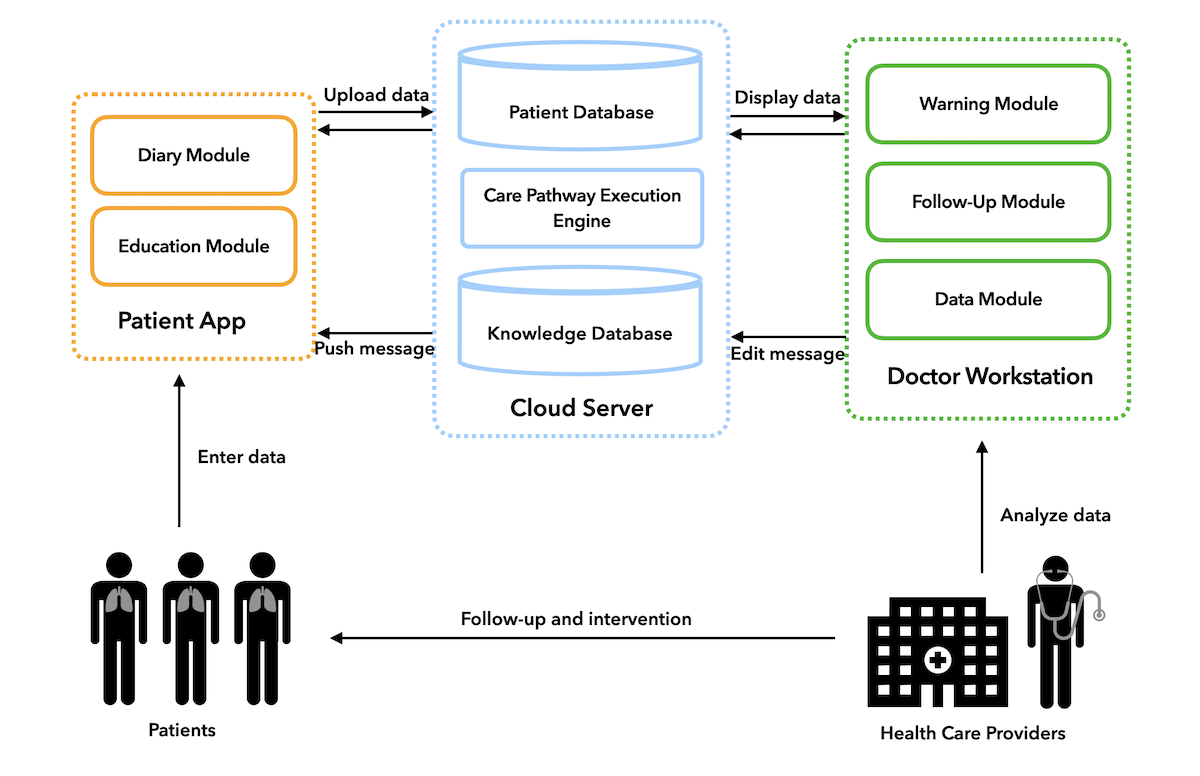
System architecture.

Based on this pathway, we implemented our mHealth-based COPD management system. As shown in Figure 2, the system contained 3 parts: patient app, doctor workstation, and cloud server. The patient app ran on a smartphone and had 2 main modules: diary module and education module. The diary module guided patients to record daily medication and symptoms. Moreover, every 2 weeks, patients were required to record their PEF value, CAT, PHQ-9, and GAD-7 scales through this module. The PEF value can be measured via a simple peak flow meter, which is available in remote Chinese areas. The education module included COPD-related educational materials in the form of videos and text. Patients received personalized materials according to their current situation [27]. We provided 2 versions of the app (Android and iOS). Both versions had the same functionality, and all the data were synchronized with the cloud.

The doctor workstation was a web-based platform for health care providers with 3 modules: warning module, follow-up module, and data module. The warning module alerted health care providers as soon as the system detected abnormal data uploaded by patients. The follow-up module provided a template for health care providers to record each follow-up in a uniform format. The data module provided complete patient information, including demographics, in-hospital examination results, and self-monitoring data history. Detailed screenshots and a function description of the patient app and doctor workstation can be found in Multimedia Appendix 2.

The cloud server securely stored all data and had an inference engine to provide decision support to the clients based on the pathway, such as patient classification, follow-up scheduling, and warning generation. In addition, it had a recommendation service for delivering educational material.

### Data Collection and Analysis

#### Primary and Secondary Outcomes

The primary outcomes included changes in PEF values; changes in QoL, as measured by the CAT scale; and changes in psychological condition, as measured by the PHQ-9 and GAD-7 scales. These outcomes reflected the COPD-specific health status of patients. As mentioned before, PEF values were measured using a peak flow meter and were uploaded via the patient app, while the 3 scales (CAT, PHQ-9, and GAD-7) were completed directly in the patient app.

The secondary outcome was the number of acute exacerbations during the study. An acute exacerbation was classified as a type of warning in the pathway and was detected from symptoms recorded by the patients. We considered dyspnea, sputum purulence, and sputum volume as major symptoms, each scored as 5 points, and nasal discharge or congestion, sore throat, cough, and wheeze as minor symptoms, each scored as 1 point, according to a previously validated definition [28]. A suspected exacerbation was automatically detected by the system if the summed symptom score was >6 points for ≥2 consecutive days. The detection required further confirmation by health care providers to ensure that the symptoms were caused by COPD.

#### Compliance

**Figure 3:**
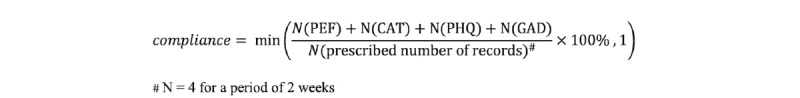
Calculation of patient compliance during a fixed period.

﻿In addition to the outcomes, patient compliance during the study was analyzed because of its relevance to self-efficacy [29] and its potential to show system availability. In this study, we defined compliance as the ratio of actual frequency of records to the prescribed number of records for the PEF, CAT scale, PHQ-9 scale, and GAD-7 scale [26], as shown in Figure 3. Patients were required to upload these 4 types of data every 2 weeks. We calculated the compliance for each month within the 6-month period.

#### Adverse Events

Information regarding serious adverse events was collected and recorded for further analysis. These events included deaths, hospitalizations, and clinic visits due to persistent respiratory symptoms. Only records related to COPD were counted. The adverse outcomes were tracked mainly in 2 ways. First, health care providers checked the hospital information system of the General Hospital of Ningxia Medical University and its affiliated hospitals regularly for records of hospital admission and clinic visits. Second, when conducting routine follow-ups, patients were asked whether they had gone to other hospitals during the study period. For the clinic visits and hospitalizations recorded for adverse outcomes, we also collected records of patients during the 6 months prior to enrollment in the study.

#### Statistical Analysis

Python 3.6 was used for data preprocessing, including extraction of clinical outcomes from the database and calculation of compliance. SPSS V23.0 was used for statistical analyses. A paired Student *t* test was used to analyze changes in PEF values. The original scores of the 3 scales before and after the 6-month study were analyzed using Wilcoxon signed-rank tests. A *P* value <.05 was considered statistically significant. Table 1 summarizes the data collected in this study.

**Table 1:** Data collection and statistical analysis.

Data			Collection time		Collection method		Statistical test
**Primary clinical outcomes**							
	PEF^a^	At baseline and after 6 months		Peak flow meter		Paired *t* test	
	CAT^b^	At baseline and after 6 months		Scale		Wilcoxon signed-rank test	
	PHQ-9^c^	At baseline and after 6 months		Scale		Wilcoxon signed-rank test	
	GAD-7^d^	At baseline and after 6 months		Scale		Wilcoxon signed-rank test	
**Secondary clinical outcome**							
	Acute exacerbation	During the study period		Detected by the system according to symptoms and confirmed by health care providers		None	
Compliance			During the study period (each month)		Calculated by the system		None
**Adverse event**							
	Death	During the study period		From HIS^e^ and follow-up		None	
	Hospitalizations	During the 6 months prior to enrollment and during the study period		From HIS and follow-up		None	
	Clinic visits	During the 6 months prior to enrollment and during the study period		From HIS and follow-up		None	

^a^PEF: peak expiratory flow.

^b^CAT: Chronic obstructive pulmonary disease Assessment Test.

^c^GAD-7: Generalized Anxiety Disorder 7.

^d^PHQ-9: Patient Health Questionnaire 9.

^e^HIS: hospital information system.

## Results

### Participant Characteristics

**Figure 4:**
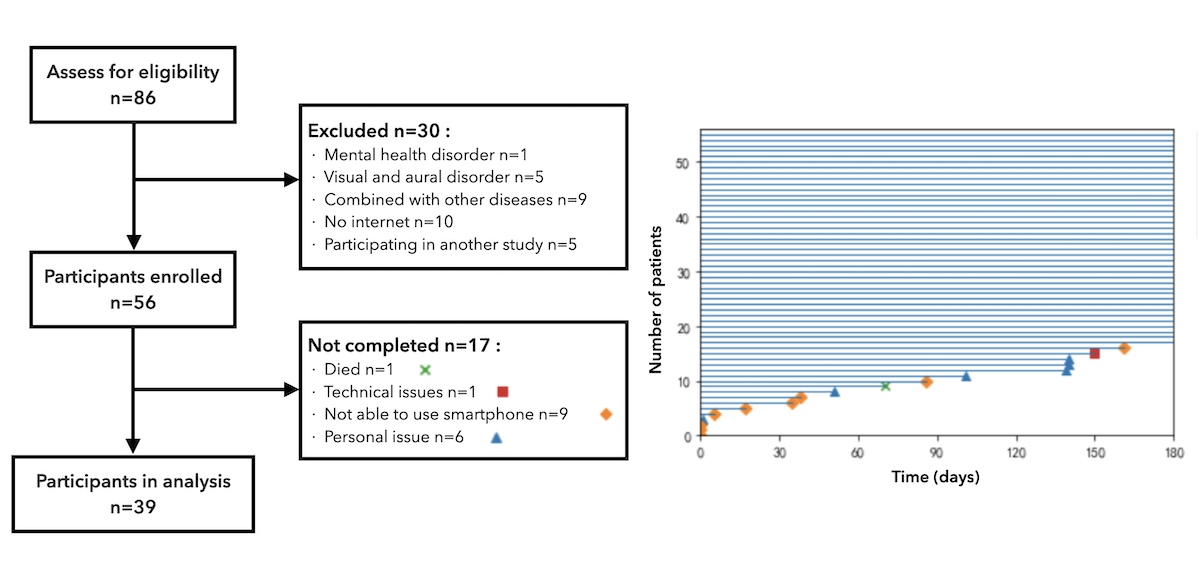
Flow diagram of patient recruitment and the study timeline.

A total of 56 patients was enrolled in the study, and 39 patients completed the study. Seventeen participants (17/56, 30%) quit the study prematurely for various reasons. Figure 4 shows the flow diagram of patient recruitment and the study timeline that demonstrates the exit point of the 17 participants.

The demographics and clinical characteristics of the 39 participants who completed the study are presented in Table 2. There were more male patients than female patients (male: 36/39, 92%; female: 3/39, 8%), and the age ranged from 49 years old to 76 years old. Approximately 85% (33/39) of the participants had an educational background of high school or below, and only 15% (6/39) had at least a college degree. A total of 33 participants were ex-smokers (30/39, 77%) or current smokers (3/39, 8%). Furthermore, the 3 current smokers quit smoking after the study. In terms of COPD severity and pulmonary function, 18% (7/39), 41% (16/39), 15% (6/39), and 26% (10/39) of the participants were at the GOLD1, GOLD2, GOLD3, or GOLD4 stage, respectively. The mean baseline forced expiratory volume in 1 second was 49.91% of predicted (95% CI, 42.54%-57.27%).

**Table 2:** Characteristics of patients who completed the study (n=39).

Patient demographic and clinical characteristics		Results
**Sex, n (%)**		
	Male	36 (92)
	Female	3 (8)
Age (years), mean (SD)		61.82 (6.09)
**Education background, n (%)**		
	Primary school and below	5 (13)
	Secondary school	12 (31)
	High school	16 (41)
	Graduate and above	6 (15)
**Smoking, n (%)**		
	Ex-smoker	30 (77)
	Current smoker	3 (8)
	Non-smoker	6 (15)
**Pulmonary function**		
	FEV1^a^ %pred (%), mean (SD)	49.91 (22.73)
	FEV1/FVC^b^ (%), mean (SD)	51.45 (13.97)
**COPD^c^ severity, n (%)**		
	GOLD1^d^	7 (18)
	GOLD2	16 (41)
	GOLD3	6 (15)
	GOLD4	10 (26)

^a^FEV1: forced expiratory volume in 1 second.

^b^FVC: forced vital capacity.

^c^COPD: chronic obstructive pulmonary disease.

^d^GOLD: The Global Initiative for Obstructive Lung Disease.

### Primary and Secondary Outcomes

Table 3 presents the clinical outcomes of the study: PEF for airflow obstruction, CAT for QoL, and PHQ-9 and GAD-7 for psychological state. The psychological state of patients who completed the study significantly improved after the 6-month intervention, especially for depression, as measured by the PHQ-9 scale (*P*=.001). The CAT score after 6 months of management was also significantly lower than that at baseline (*P*=.003). In terms of PEF, the mean value decreased after management but was not significant (*P*=.11). From a clinical viewpoint, the median CAT score remained at a medium level (11-20 [5]), and the median GAD-7 score remained at the “no anxiety disorder” level (0-4 [26]) before and after the study. The median PHQ-9 score changed from mild depression (5-9 [25,30]) to minimal depression (0-4 [25]).

**Table 3:** Clinical outcomes before and after the study for patients who completed the study (n=39).

Outcomes	Baseline	After 6 months	*P* value
PEF^a^, mean (SD)	366.1 (106.7)	313.1 (116.6)	.11
CAT^b^, median (Q25^c^-Q75^d^)	17.0 (14.0-23.0)	14 (10.0-18.0)	.003
PHQ-9^e^, median (Q25-Q75)	6.0 (3.0-9.0)	4.0 (0.0-6.0)	.001
GAD-7^f^, median (Q25-Q75)	4.0 (1.0-6.0)	3.0 (0.0-6.0)	.01

^a^PEF: peak expiratory flow.

^b^CAT: COPD Assessment Test.

^c^Q25: first quartile.

^d^Q75: third quartile.

^e^PHQ-9: Patient Health Questionnaire 9.

^f^GAD-7: Generalized Anxiety Disorder 7.

In terms of acute exacerbations, 459 warning events were detected by our system during the study, 117 of which were acute exacerbation warnings related to symptoms. Among these symptom warning events, 7 were excluded due to worsening symptoms caused by cardiovascular disease and other diseases, and only 110 events were confirmed as exacerbation of COPD by health care providers (median 2.0 per 6 months, IQR 1.0-5.0). Moreover, 64.5% (71/110) of these exacerbations ended up as a clinic visit or hospitalization. The detailed number of acute exacerbations for each patient can be found in Multimedia Appendix 3.

### Compliance

**Figure 5:**
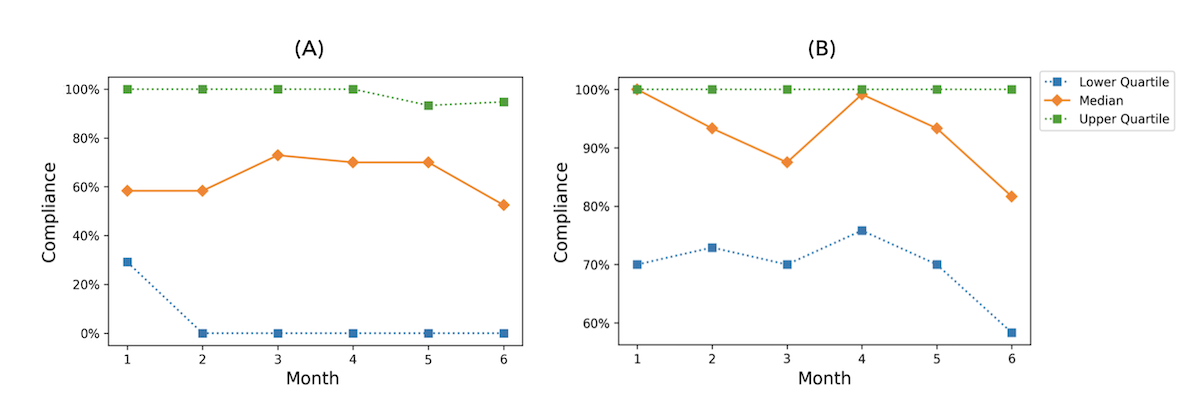
Quartiles of and median participant compliance per month over the 6-month period for (A) all enrolled patients (n=56) and (B) those who completed the study (n=39).

Figure 5 shows the participant compliance per month for all enrolled patients (n=56) and patients who completed the study (n=39). Some participants recorded data more often than prescribed; thus, their compliance was over 100% but was calculated as 100%. Because more than one-quarter of participants withdrew midway, the lower quartile in Figure 5A tended to be 0%. For those who completed the study (n=39), monthly compliance remained at a high level, as shown in Figure 5B. We also calculated the overall compliance of these patients during the 6-month period. The median was 91.1%, (IQR 67%-100%). The overall trends of the uploaded data can also be found in Multimedia Appendix 3.

### Adverse Events

According to our definition, a total of 72 adverse events occurred during the study, including 1 death, 52 clinic visits, and 19 hospitalizations. We compared the number of hospitalizations and clinic visits for each patient before and after the study (see Multimedia Appendix 3 for detailed results). During the 6 months prior to enrollment, patients (n=39) reported a total of 115 clinic visits and 69 hospitalizations related to COPD. Both clinic visits (decreased of 54.8%) and hospitalizations (decrease of 72.5%) decreased over the 6-month period compared with the same period before the study.

## Discussion

### Principal Findings

In this study, we designed and implemented a feasible method to manage COPD in Chinese remote areas using an mHealth-based system over a 6-month period, despite the challenges with pulmonary function monitoring outside of the hospital. Although pulmonary function did not significantly change with the implementation of this system, we found an improvement in COPD-specific QoL and mental health assessments of participants.

Some of our results could be attributed to the diversity of COPD severity among participants. In terms of acute exacerbation, a total of 110 exacerbations w identified during the study, because COPD severity for >80% of participants was at least a GOLD2 level. The median number of exacerbations (2.0 per 6 months) was close to the result (3.0 per year) from a previous survey conducted in the Asia-Pacific region [31]. According to the records of adverse events, over half of these exacerbation cases led to a clinic visit or hospitalization. The rest of the exacerbations were handled remotely by health care providers. The high variability in COPD severity also explains the large SDs of the PEF values.

Of the 56 patients, 17 patients quit midway through the study, mainly due to difficulty using smartphones. The relatively high dropout rate could be attributed to the low educational attainment of participants (about 85% had achieved an educational level of high school or below). For patients who completed the study (n=39), the overall median 6-month compliance reached 91.1%, which was relatively high compared with the rates in prior studies [11,32]. In addition, after the 6-month intervention, 3 current smokers quit smoking. These results indicate that, despite a low educational level, our pathway-driven, timely intervention played an important role in behavioral change by COPD patients.

### Comparison With Prior Work

To understand the innovation of this study, we compared our study with prior work from multiple aspects. In terms of QoL, significant improvement in COPD-specific QoL was observed in this study, whereas prior studies reported no change in QoL or only changes in general health status [11,32-35]. CAT and EuroQol-5D (EQ-5D) [36] are the most popular scales for COPD management [11,32-35]. CAT is more disease-specific, whereas EQ-5D focuses more on general health status. Studies using the EQ-5D for QoL measurement often introduced other scales as a supplement for COPD-specific symptoms [11,32]. More scales capture more details for research but place an extra burden on patients, which may lead to poor compliance. Moreover, previous research has shown that the CAT is more suitable for the comprehensive assessment of COPD severity [37]. Therefore, we chose the CAT as the only outcome for QoL.

In terms of pulmonary function, prior studies [33,37,38] did not directly compare changes in pulmonary function measured via portable devices at home. In this study, we chose PEF for continuous pulmonary function monitoring, and the PEF values showed a downward trend because of the irreversible decline of pulmonary function. Although portable spirometers are available and have been used in some studies [33,35,38], mass promotion is difficult in China considering the economic and population factors. Equipped with a peak flow meter and an mHealth system, PEF is easy to measure at home and under supervision. Moreover, to some degree, PEF measurement alone cannot be reliably used as the only diagnostic test because of its weak specificity [5]; hence, we combined PEF with CAT and symptoms to evaluate COPD-specific health status and monitor acute exacerbations. Our study showed that elderly people with a low educational background could master PEF measurements. With limited medical resources, PEF measured by a peak flow meter is suitable for COPD management in Chinese remote areas.

In terms of psychological conditions, except for our study, only 1 study [34] found significant improvement in psychological distress with a long-term (12-month) telehealth intervention but without a clear explanation. Similar to the studies by Farmer et al [11] and Rixon et al [34], we used independent psychological scales, whereas other studies investigated psychological factors within general health status such as with the EQ-5D. In this study, the improvement in psychological status may be explained by the high frequency of communication between patients and health care providers. The interval of routine follow-up visits in the study by Farmer et al [11] was 3 months, and extra contact only occurred for safety alerts, whereas in our study, follow-ups were arranged at least every 2 weeks. With the assistance of WeChat, the communication frequency was even higher in practice.

In terms of the intervention, the care pathway was a unique strategy that we proposed in the field of COPD management, breaking the heavy management tasks into detailed pieces that can be quantified and executed by computers. Combined with mHealth, it was simple and convenient to deliver the COPD management system driven by the care pathway. With the help of this system, both patients and health care providers were aware of what exactly to do in different phases of management. Although the pathway is not yet perfect, it could improve the outcome of patients and efficiency of health care providers.

### Strengths and Limitations

Our study has several strengths. First, we defined compliance management as part of the care pathway. An extra follow-up would be required for patients with local compliance. In this study, inspired by our previous study [39], we calculated patient compliance according to the frequency of their self-monitoring behaviors. Compared with prior studies [11,32], our compliance was more objective and practical over the long term. According to our results, the monthly compliance remained high for patients who completed the study. Second, WeChat played a surprisingly important role in our study. WeChat was first designed as a follow-up tool, but as the study progressed, patients found it a convenient way of communication and to have their questions answered. The WeChat chat group produced dozens of messages every day during the study. Patients said that they could learn a lot about self-management skills in the group by just viewing messages because many common questions were raised and answered immediately. To some extent, getting feedback in a short time helped patients build trust with health care providers and maintain high compliance. From another point of view, group talking introduced social activity for COPD patients, and doctors could intervene psychologically by managing patients’ mental health and encouraging group members. This is a convincing reason for the mental health improvement in our study. The impact of social apps on COPD management may be worthy of further study.

Numerous potential weaknesses need to be acknowledged. First, the study was limited by the sample size and gender imbalance. The number of participants was relatively small (n=39), while 92% of the participants were male. The gender imbalance was mainly due to the higher incidence of COPD in men than in women in Ningxia [4]. The effect of our system on a larger scale and female patients needs further investigation. Second, when collecting the records from clinic visits and hospitalizations, an inherent bias existed because patients might go to hospitals for all related symptoms prior to the study due to a lack of communication with health care providers. Therefore, the difference in pre-study and post-study records may not reflect the change in disease condition. Moreover, from the methodological perspective, our pilot observational study could not yield the highest level of evidence, and a randomized controlled trial should be performed for further study.

### Future Work

In future work, we will include more female patients and conduct a high evidence-level clinical trial. A comparison test should be conducted to determine if our intervention can perform well on a larger scale. We also plan to investigate the actual effect of WeChat in improving patient outcomes through the trial. The satisfaction rate of patients using WeChat may be presented as an outcome. Another direction for future work is to explore a more intelligent care pathway to provide more individualized and precise care for patients with COPD. The feasibility of applying a data-driven artificial intelligence approach to strengthen the current pathway will be investigated.

### Conclusions

We introduced an mHealth-based method for COPD management at the community level. The tailored closed-loop care pathway was found to be feasible and effective in areas with limited medical resources. Although the decrease in pulmonary function was irreversible, patients’ COPD-specific QoL and psychological status improved significantly after our 6-month study. Moreover, despite the relatively low educational background of participants, proper education and frequent communication may encourage their acceptance and use of smartphones to support COPD self-management. Researchers should choose the most appropriate intervention strategy according to the actual situation of the intervention area.

## Appendix

Multimedia Appendix 1 Detailed description of the closed loop care pathway.

Multimedia Appendix 2 Detailed screenshots and function list of the mHealth system.

Multimedia Appendix 3 Detailed results of primary and secondary outcomes.

## Abbreviations

CAT: COPD Assessment Test
COPD: chronic obstructive pulmonary disease
EQ-5D: EuroQol-5D
FEV1: forced expiratory volume in 1 second
FVC: forced vital capacity
GAD-7: Generalized Anxiety Disorder-7
GOLD: Global Initiative for Chronic Obstructive Lung Disease
HIS: hospital information system
mHealth: mobile health
PEF: peak expiratory flow
PFT: pulmonary function test
PHQ-9: Patient Health Questionnaire 9
QoL: quality of life
