# Supplementary material for: Using Mobile Health Technology to Deliver a Community-Based Closed-Loop Management System for Chronic Obstructive Pulmonary Disease Patients in Remote Areas of China: Development and Prospective Observational Study
Source: JMIR Mhealth Uhealth. 2020 Nov 25;8(11):e15978. doi: 10.2196/15978 (PMC7725649; doi:10.2196/15978)
Supplement: Multimedia Appendix 1 [file mhealth_v8i11e15978_app1.pdf]

Closed-loop Care Pathway Design

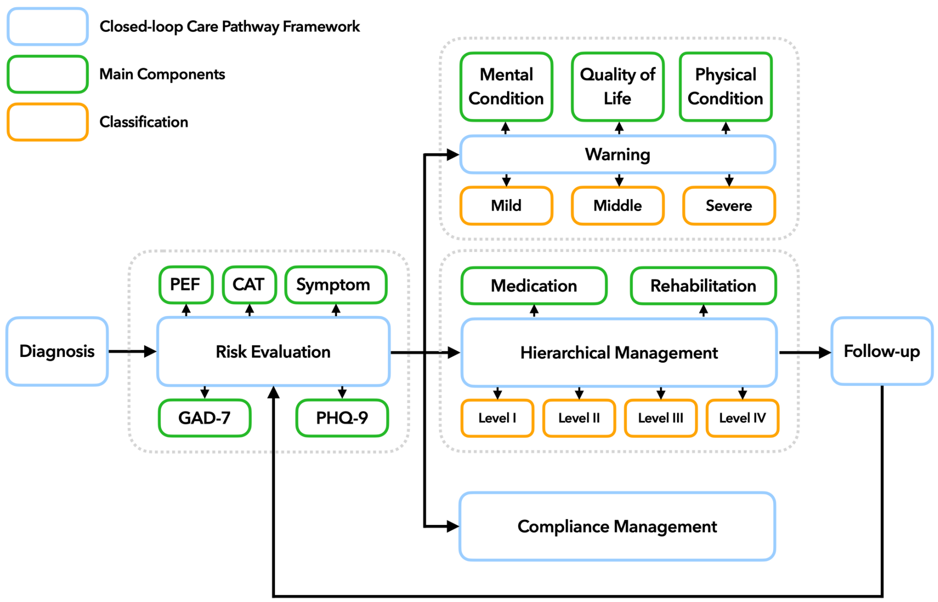

COPD Risk Evaluation

In this study, we conducted a comprehensive assessment of patients conditions with exacerbation risk (symptoms), quality of life (COPD Assessment Test, CAT [1]), mental health (Patient Health Questionnaire-9 scale, PHQ-9 [2], and Generalized Anxiety Disorder 7 scale, GAD-7 [3]) and pulmonary function (peak expiratory flow, PEF[4-6]) every two weeks and patients were classified into 4 levels as Supplementary Table 1. Patients who satisfy all five conditions are evaluated as level I. If any one of five condition meets the threshold of level II, III or IV, patient will be classified into this particular level.

Supplementary Table 1. Risk Evaluation*

Evaluation Level	Symptoms	CAT[a]	PEF%Pred[b]	PHQ-9[c]	GAD-7[d]
**I**	None	≤ 10	≥ 80%	≤ 4	≤ 4
**II**	Wheeze, cough	11-20	60-80%	5-9	5-9
**III**	Sputum purulence, dyspnea	21-30	60-80%	10-14	10-14
**IV**	Fever, drowsiness	>30	< 60%	15-27	15-21

[a]CAT: COPD Assessment Test
[b]PEF % Pred: peak expiratory flow % Prediction
[c]GAD-7: Generalized Anxiety Disorder 7

[d]PHQ-9: Patient Health Questionnaire 9
*The standard of CAT, PHQ-9 and GAD-7 warnings are defined according to the origin paper or guideline of the three scales.

Hierarchical Management

According to the results of evaluation, patients will receive appropriate intervention and follow-up accordingly, which is called hierarchical management, mainly including medication and pulmonary rehabilitation. Patients in different levels will receive a tailored two-stage medication plan as shown in Supplementary Figure 2. The medication plans are referred to GOLD guidelines and doctor's consent is necessary for final prescription. Hospital admission is recommended if patients remain unstable after switching to second medication plan. Patients are asked to perform proper pulmonary rehabilitation based on evaluation level. Videos are made for each kind of rehabilitation exercise by our nurses, demonstrating the details step by step.

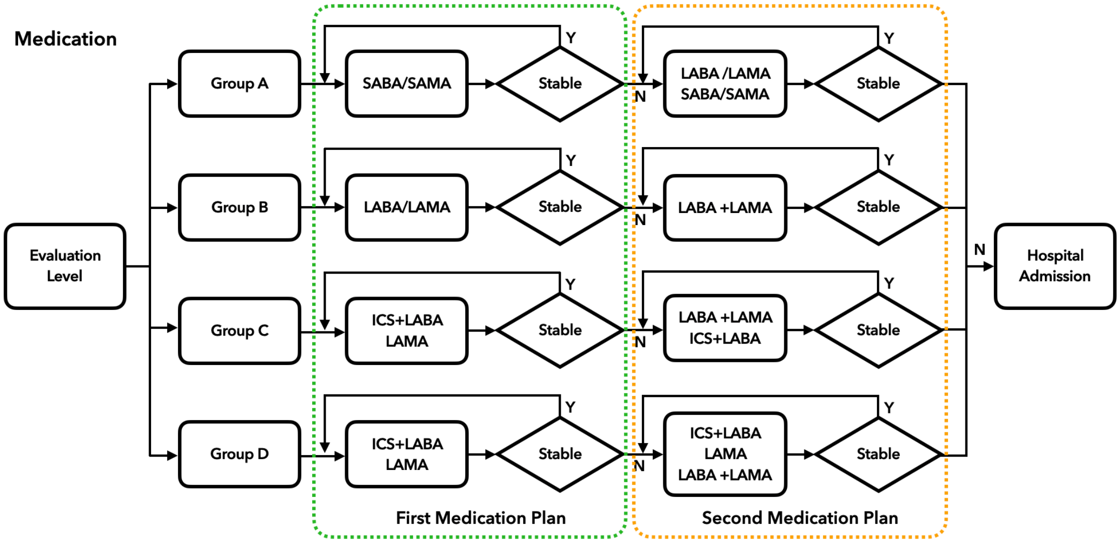

Supplementary Figure 2. Medication of Hierarchical management

Supplementary Table 2. pulmonary rehabilitation of Hierarchical management

Evaluation Level		I	II	III	IV
Limb exercise		+	+	+	+
Pursed lips breathing		+	+	+	+
Abdominal breathing		+	+	+	+
Breathing exercise	Seated	+	+	+	-
	Stood	+	+	-	-
	Laying	+	+	+	-

**Oxygen therapy**	If patients have indications for oxygen therapy*

*Note: A $PaO_2 \leq 55$mmHg or a PaO_2 of 56-59mmHg when there is also evidence of end-organ dysfunction secondary to chronic hypoxia.

Warning

Warnings were defined in Supplementary Table 3, which works as reference for the condition analysis by healthcare providers. The standard of CAT, PHQ-9 and GAD-7 warnings are defined according to the original paper or guideline of the three scales. The two thresholds of PEF warning—60% and 80% of standard value—are proposed by this study, where the standard value is referred to the authoritative research of Chinese pulmonary function [7].

Supplementary Table 3. Warning

Warning	PEF/Pred[a]	CAT[b]	PHQ-9[c]	GAD-7[d]
Mild	60-80%	11-20	10-14	5-9
Middle	60-80%	21-30	15-19	10-14
Severe	<60%	31-40	20-27	15-21

[a]PEF % Pred: peak expiratory flow % Prediction
[b]CAT: COPD Assessment Test
[c]GAD-7: Generalized Anxiety Disorder 7
[d]PHQ-9: Patient Health Questionnaire 9

Compliance Management

Patient compliance will be calculated every month by the system. In this study, we define the compliance as the ratio of actual frequency of records to the prescribed number of records for PEF, CAT scale, PHQ-9 scale and GAD-7 scale. Patients are required to upload these four types of data every two weeks. Follow-up is performed as the main intervention to maintain and improve patient compliance. An extra follow-up will be arranged for low compliance.

$$compliance = \frac{N(\text{PEF}) + \text{N}(\text{CAT}) + \text{N}(\text{PHQ}) + \text{N}(\text{GAD})}{N\text{（prescribed number of records）}} \times 100\%$$

This part has been introduced in the data collection of manuscript.

Follow-up

Generally, follow-up is conducted every two weeks. In the event of a warning or low compliance, an extra follow-up will be arranged. Telephone is a traditional tool to perform follow-up but sometimes it is inefficient because patients may be inconvenient to answer the call or do not want to be disturbed. Therefore, we introduced WeChat, the most popular instant messaging app in China, as an alternative follow-up method for patients. This part has been introduced in the study design of manuscript

Reference

1. Jones P, Harding G, Berry P, et al. Development and first validation of the COPD Assessment Test. European Respiratory Journal, 2009;34(3): 648-654. PMID: 19720809
2. Kroenke K, Spitzer R L. The PHQ-9: a new depression diagnostic and severity measure. Psychiatric annals, 2002;32(9): 509-515. PMID: 11556941
3. Spitzer R L, Kroenke K, Williams J B, et al. A brief measure for assessing generalized anxiety disorder: the GAD-7. Archives of internal medicine, 2006;166(10): 1092-1097. PMID: 16717171
4. Quanjer P, Lebowitz M, Gregg I, et al. Peak expiratory flow: conclusions and recommendations of a Working Party of the European Respiratory Society. European respiratory journal, 1997;10(24): 2s. PMID: 9098701
5. Jackson H. Detecting chronic obstructive pulmonary disease using peak flow rate: cross sectional survey. BMJ Open, 2003;327(7416): 653-654. PMID: 14500437
6. Casale R P P. Cosinor analysis of circadian peak expiratory flow variability in normal subjects, passive smokers, heavy smokers, patients with chronic obstructive pulmonary disease and patients with interstitial lung disease. Respiration, 1997;64(4): 251-6. PMID: 9257358
7. Zhong N, Wang C, Yao W, et al. Prevalence of chronic obstructive pulmonary disease in China: a large, population-based survey. Am J Respir Crit Care Med. 2007;176(8):753-760. PMID:17575095
