# Supplementary material for: Using Mobile Health Technology to Deliver a Community-Based Closed-Loop Management System for Chronic Obstructive Pulmonary Disease Patients in Remote Areas of China: Development and Prospective Observational Study
Source: JMIR Mhealth Uhealth. 2020 Nov 25;8(11):e15978. doi: 10.2196/15978 (PMC7725649; doi:10.2196/15978)
Supplement: Multimedia Appendix 2 [file mhealth_v8i11e15978_app2.pdf]

mHealth System Description

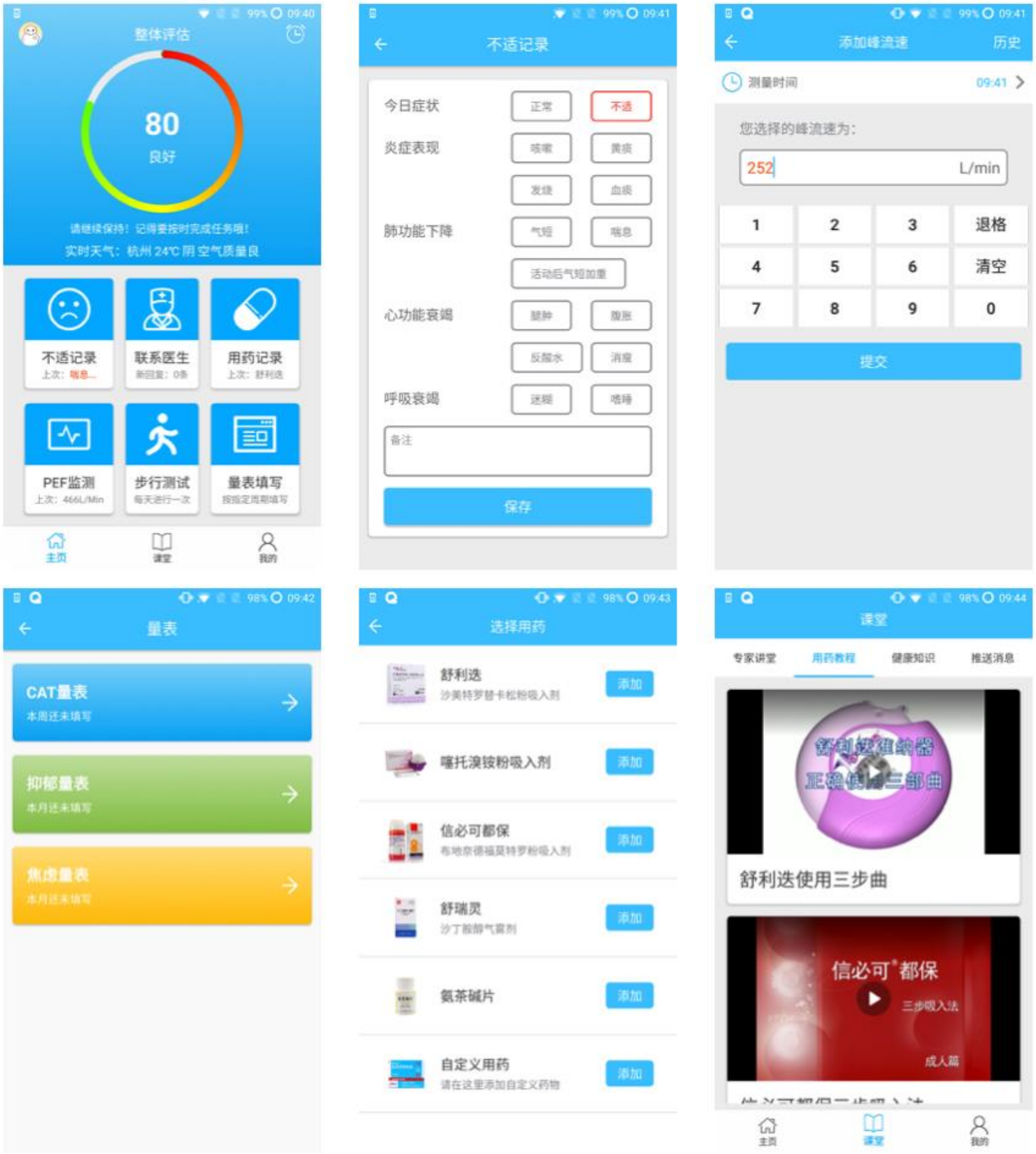

Supplementary Figure 1 Snapshots of Patient app

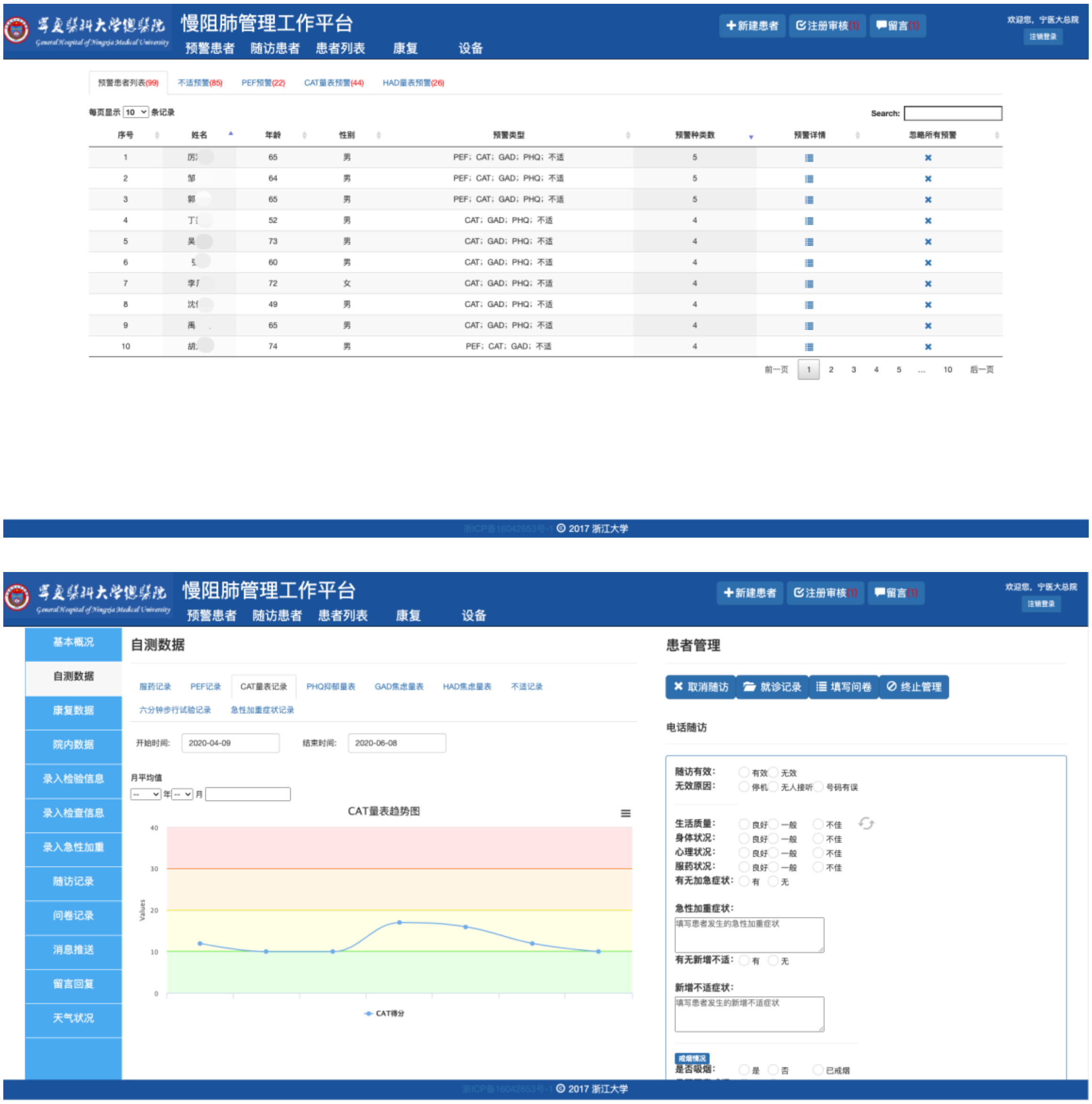

Supplementary Figure 2 Snapshots of Doctor workstation

Supplementary Table 1 Function list of mHealth System in this study

Functions	Descriptions
**Patient app**	
**Diary module**	
PEF[a] value record	Used to record PEF values.
CAT[b] scale record	Used to fill out CAT scale and record the result.
PHQ-9[c] scale record	Used to fill out these two scales and record results.
GAD-7[d] scale record	
Medication record	Used to record daily medication.
Symptom	Patient can select the corresponding symptoms if he feels uncomfortable.

		6-minute walk test record	Patient could take a 6-minute walk test according to the app's instructions and the test result will be automatically measured via GPS and saved.
		Local air quality data	Record data provided by www.heweather.com every day for each participant based on their address.
	**Education module**		
		Knowledge	This app provides videos and texts associated with inhalants guidance, pulmonary rehabilitation, smoke cessation and other COPD-related knowledge or health tips.[e]
		Communication	Patients will receive personalized materials according to their current situation
**Doctor workstation**			
	**Warning module**		
		PEF value warning	Detect and alert abnormal data uploaded by patients.
		CAT scale warning	
		PHQ-9 scale warning	
		GAD-7 scale warning	
		Symptom	
	**Follow-up module**		
		Follow-up	Used to record the follow-up context in a uniform format.
		Communication	Send messages or materials to patients.
	**Data module**		
		Demographic information	Including patient name, gender, age, height, weight, et al.
		In-hospital laboratory and examination results	Including blood routine test, blood biochemical test, blood gas analysis data, pulmonary function test, CT and ultrasonography from the EMR system in the hospital.
		Self-management data	All the data uploaded via patient app. The data is presented in the form of tables and graphs.
**Cloud server**			
		Data store	Store all the data securely.
		Inference engine	Providing decision support.
		Recommendation service	Delivering educational materials.

[a]PEF: peak expiratory flow

[b]CAT: COPD Assessment Test

[c]GAD-7: Generalized Anxiety Disorder 7

[d]PHQ-9: Patient Health Questionnaire 9

[e] The behavior including reading health knowledge and watching educational videos were caught and uploaded automatically.
