# Supplementary material for: Using Mobile Health Technology to Deliver a Community-Based Closed-Loop Management System for Chronic Obstructive Pulmonary Disease Patients in Remote Areas of China: Development and Prospective Observational Study
Source: JMIR Mhealth Uhealth. 2020 Nov 25;8(11):e15978. doi: 10.2196/15978 (PMC7725649; doi:10.2196/15978)
Supplement: Multimedia Appendix 3 [file mhealth_v8i11e15978_app3.pdf]

Details of Outcomes

Supplementary Table 1 Details of warning events, exacerbations, hospitalization, and clinic visit.

Patient number	Hospitalization, n/6 months		Clinic visits, n/6 months		Warning events, n	AE, n
	before	after	before	after		
1	1	0	0	0	1	1
2	4	0	6	2	34	4
3	0	0	3	1	0	2
4	2	0	1	0	0	1
5	3	2	5	3	73	7
6	1	0	4	3	0	3
7	3	1	0	1	1	2
8	2	0	0	0	9	3
9	3	1	0	0	12	3
10	0	0	2	0	0	1
11	2	0	0	0	0	1
12	3	1	0	6	27	7
13	3	2	0	0	74	5
14	0	0	1	0	1	1
15	3	0	10	9	0	9
16	2	0	3	2	2	3
17	0	0	2	1	0	1
18	4	2	4	3	55	5
19	4	2	0	0	4	2
20	0	0	3	1	0	2
21	0	0	3	2	80	5
22	2	0	2	0	0	1
23	2	0	2	2	0	2
24	2	0	3	0	0	1
25	0	0	1	0	2	2
26	3	1	10	3	0	4
27	1	0	8	2	19	5
28	0	0	0	0	0	0
29	3	0	7	6	5	6
30	3	0	6	3	50	5
31	2	0	4	0	1	2
32	1	0	2	0	0	1
33	3	5	6	0	1	5
34	3	0	9	0	5	1
35	1	0	5	1	0	1
36	1	1	0	0	2	1
37	0	0	2	1	0	1
38	1	0	1	0	1	1
39	1	1	0	0	0	3
Total	69	19	115	52	459	110

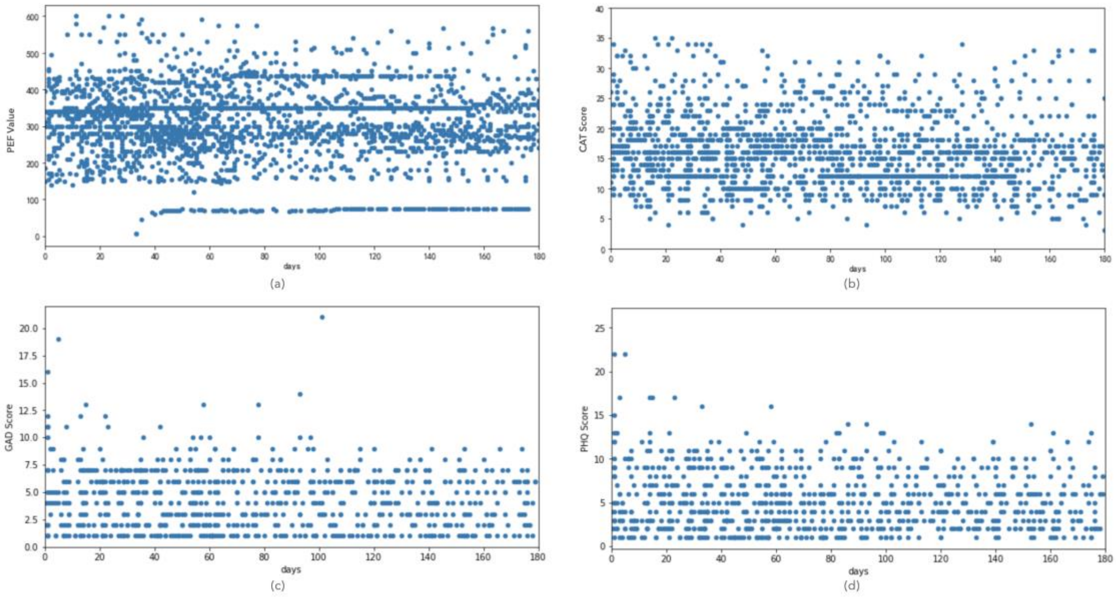

Supplementary Figure 1 Data records from patients during this study. (a) Records for the PEF. (b) Records for the CAT scale. (C) Records for the GAD-7 scale. (d) Records for the PHQ-9 scale.
